# A population pharmacokinetic model for simvastatin and its metabolites in children and adolescents

**DOI:** 10.1007/s00228-019-02697-y

**Published:** 2019-06-06

**Authors:** Kayode Ogungbenro, Jonathan B. Wagner, Susan Abdel-Rahman, J. Steven Leeder, Aleksandra Galetin

**Affiliations:** 1 Centre for Applied Pharmacokinetic Research, Division of Pharmacy and Optometry, School of Health Sciences, Faculty of Biology, Medicine and Health, Manchester Academic Health Science Centre, University of Manchester, Manchester, M13 9PT UK; 2 Ward Family Heart Center, Children’s Mercy Kansas City, Kansas City, MO USA; 3 Division of Clinical Pharmacology, Toxicology and Therapeutic Innovation, Children’s Mercy Kansas City, Kansas City, MO USA; 4 Department of Pediatrics, School of Medicine, University of Missouri-Kansas City, Kansas City, MO USA

**Keywords:** Simvastatin, Population pharmacokinetics, Children and adolescents, Metabolites, Modelling

## Abstract

**Purpose:**

Poor adherence to dietary/behaviour modifications as interventions for hypercholesterolemia in paediatric patients often necessitates the initiation of statin therapy. The aim of this study was to develop a joint population pharmacokinetic model for simvastatin and four metabolites in children and adolescents to investigate sources of variability in simvastatin acid exposure in this patient population, in addition to *SLCO1B1* genotype status.

**Methods:**

Plasma concentrations of simvastatin and its four metabolites, demographic and polymorphism data for OATP1B1 and CYP3A5 were analysed utilising a population pharmacokinetic modelling approach from an existing single oral dose (10 mg < 17 years and 20 mg ≥ 18 years) pharmacokinetic dataset of 32 children and adolescents.

**Results:**

The population PK model included a one compartment disposition model for simvastatin with irregular oral absorption described by two parallel absorption processes each consisting of sequential zero and first-order processes. The data for each metabolite were described by a one-compartment disposition model with the formation and elimination apparent parameters estimated. The model confirmed the statistically significant effect of c.521T>C (rs4149056) on the pharmacokinetics of the active metabolite simvastatin acid in children/adolescents, consistent with adult data. In addition, age was identified as a covariate affecting elimination clearances of 6-hydroxymethyl simvastatin acid and 3, 5 dihydrodiol simvastatin metabolites.

**Conclusion:**

The model developed describes the pharmacokinetics of simvastatin and its metabolites in children/adolescents capturing the effects of both c.521T>C and age on variability in exposure in this patient population. This joint simvastatin metabolite model is envisaged to facilitate optimisation of simvastatin dosing in children/adolescents.

**Electronic supplementary material:**

The online version of this article (10.1007/s00228-019-02697-y) contains supplementary material, which is available to authorized users.

## Introduction

Simvastatin (SV) is a 3-hydroxy-3methyl-glutaryl coenzyme A (HMG-CoA) reductase inhibitor licensed for the treatment of lipid disorders including hypercholesterolemia [1]. Worldwide, statins are commonly prescribed and have proven to be effective in global reduction of risk factors related to major cardiovascular events [2, 3]. Simvastatin is considered to be safe and well tolerated; however, skeletal muscle toxicity, ranging from myalgia to rhabdomyolysis, can lead to significant morbidity and mortality [4].

SV pharmacokinetics (PK) is complex; it is administered as an inactive lactone prodrug that is converted to the active form simvastatin acid (SVA) by hydrolysis or enzymatically by carboxylesterases (in the liver and small intestine) and by paraoxonases in plasma [5, 6]. SVA can also be converted back to SV via an acyl-glucuronide intermediate. In adults, both SV and SVA are extensively metabolised by CYP3A4/5 to form 6-hydroxymethyl SV (HMSV) and 6-hydroxymethyl SVA (HMSVA), respectively [6, 7]. While HMSV can be further metabolised to form HMSVA, HMSVA can also be back-converted to HMSV [6]. In addition to SVA, other acid metabolites (such as HMSVA) have been reported to possess pharmacological HMG-CoA reductase activity [8].

The genetic polymorphisms in a non-synonymous single-nucleotide (SNP), rs4149056 (*SLCO1B1* c.521T>C), coding for hepatic uptake organic anion transporting polypeptide, OATP1B1, has been reported to markedly increase systemic exposure of SVA in adults and is therefore a risk factor for muscle toxicity [9–11]. Consequently, a guideline for dosing SV when c.521T>C genotype is available has been proposed, with the recommendation of a reduction in dose or an alternative statin for variant allele patients [10].

Tsamandouras et al. [5] developed a joint population PK model for SV and SVA in adults that incorporated multiple genetic polymorphisms and clinical/demographic factors as covariates. This model confirmed clinically known effects of genetic variants (c.521T>C) and established associations between other genetic variants such as rs776746 (CYP3A5), rs12422149 (*SLCO2B1*), rs2231142 (ABCG2), rs4148162 (ABCG2), rs4253728 (PPARA), and rs35599367 (CYP3A4) and SV and SVA pharmacokinetic parameters. It also highlighted combinations of risk factors important for the PK of either SV and/or SVA that can explain the myopathy risk beyond c.521CC genotype.

As with adults, statins represent the mainstay of hypercholesterolemia treatment when lifestyle modifications fail in children [12]. With enhanced screening of hypercholesterolemia during childhood, and the known challenges with adherence to behavioural/dietary modifications, paediatric statin use is increasing [13]. Despite generally recognised differences in drug disposition in the growing child relative to adult, current paediatric SV dosage recommendations are extrapolated from existing adult data [14]. In a recent clinical study in children/adolescent [15], non-compartmental analysis of the data showed that each copy of the *SLCO1B1* c.521C allele was associated with a 2.5-fold increase in SVA systemic exposure, an effect that was more pronounced than reported in adult studies. More importantly, the 9- to 10-fold range of AUC values noted within the c.521TT and c.521TC *SLCO1B1* genotype groups exceeded the between-group variability, implying that additional factors may contribute to inter-individual variability in SVA systemic exposure in children and adolescents. In addition, 25% of the participants in the cohort were reported to have negligible SVA exposure. Using the data from Wagner et al. [15], the current work extends the analysis to the development of a population PK model to allow more comprehensive characterisation of SV and its four metabolites in children and adolescents. Simultaneous modelling of SV, SVA, and additional two hydroxymethyl and one dihydrodiol metabolites aimed to investigate sources of variability contributing to systemic exposure of both parent drug and metabolites in children and adolescents and, in particular, to provide insights into the observed high variability in the dose-exposure relationship within c.521TC genotype groups and low/undetectable concentrations of SVA in some patients.

## Methods

### Data description

The data for this analysis were obtained from a clinical study conducted at Children’s Mercy Hospital, Kansas City [15]. All patients gave written informed consent. The protocol for the study was approved by the Children’s Mercy Hospital Institutional Review Board, in accordance with appropriate regulatory and Good Clinical Practice guidelines and following ethical principles as described in the Declaration of Helsinki. Details of the study design and demographic details of the study participants were provided in the original publication [15]. The study was a single-centre, open-label, single-dose (oral) genotype-stratified study of SV (10 mg 8–17 years; 20 mg ≥ 18 years) in 32 hyperlipidemic children and adolescents including reference c.521TT genotype individuals (*n* = 15) as well as one (*n* = 15) or two (*n* = 2) c.521T>C variant allele individuals. Serial venous blood samples were obtained from participants at the following time points: 0, 0.5, 1, 1.5, 2, 3, 4, 6, and 8 h. Plasma concentrations of SV and four other metabolites, i.e. SVA, HMSV, HMSVA, and 3, 5 dihydrodiol simvastatin (DHSV), were measured using a validated ultra-high-pressure liquid chromatography–tandem mass spectrometric method [15]. The lower limit of quantification (LLOQ) for SV and the metabolites was 0.5 nM. Individual deoxyribonucleic acid (DNA) samples were also genotyped for SNPs for *SLCO1B1* (11187 G>A – rs4149015, c.388A>G – rs2306283, and c.521T>C – rs4149056) and CYP3A5 (CYP3A5*1D – rs15524, CYP3A5*3 – rs776746, and CYP3A5*6 – rs10264272) genes. Summary of the data, the total number of samples, and the fraction below the LLOQ for each analyte are in the Online Resource.

### The structural and statistical model

**Fig. 1:**
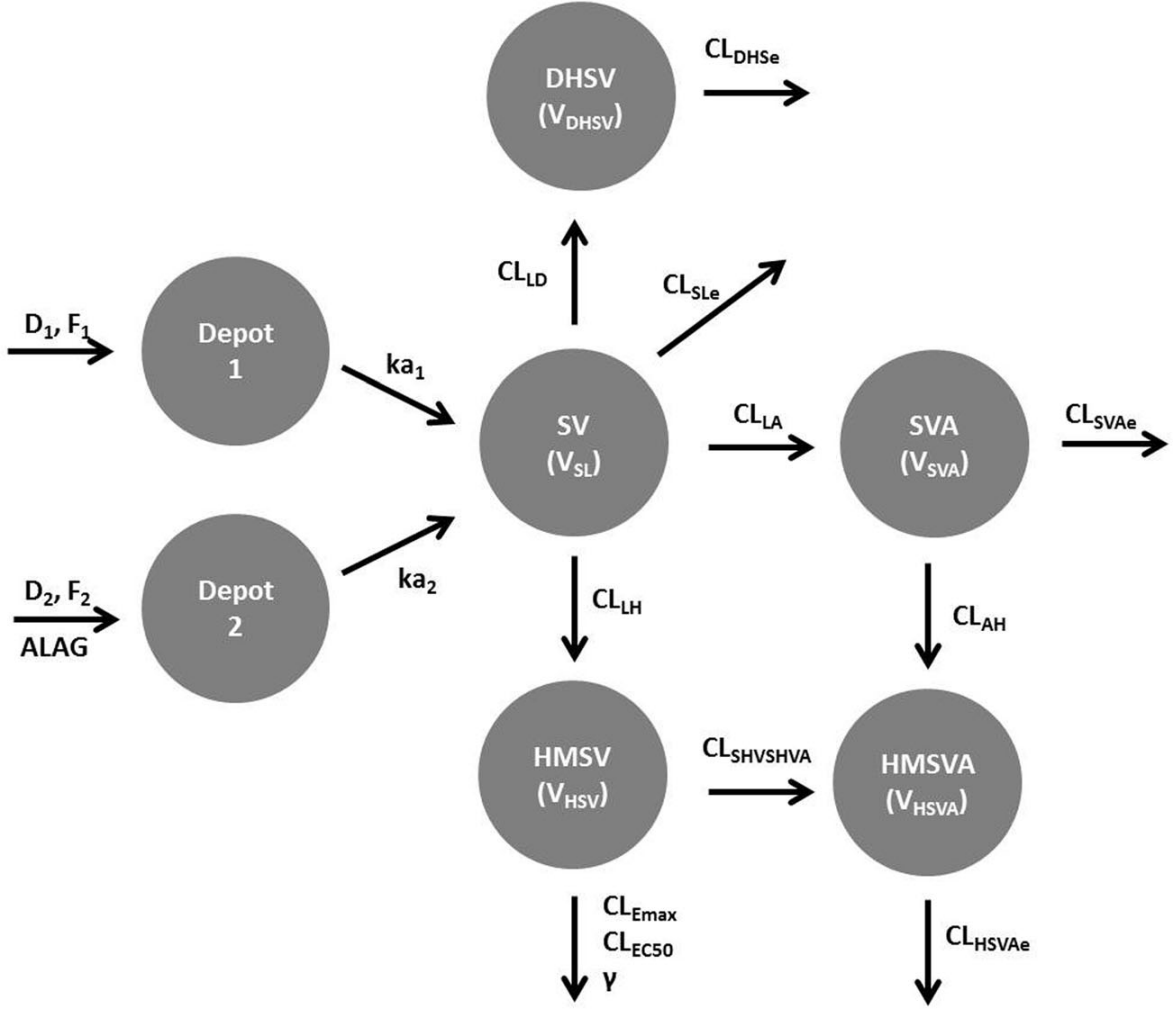
Structure of the joint model for simvastatin (SV), simvastatin acid (SVA), 6 hydroxymethyl simvastatin (HMSV), 6 hydroxymethyl simvastatin acid (HMSVA), and 3, 5 dihydrodiol simvastatin (DHSV)

A population PK model was developed for SV and the metabolites using non-linear mixed-effects modelling technique in NONMEM software (v7.4, ICON Development Solutions, Ellicott City, MD, USA) with a first-order conditional estimation method with interaction option [16]. Data exploration, output analysis, and goodness-of-fit (GOF) plots were performed in MATLAB software [17]. The available plasma concentration data for SV and four metabolites were log-transformed for simultaneous analysis. Absorption of SV was modelled using two parallel absorption processes, separated by a lag time, each consisting of sequential zero- and first-order processes. This approach was based on a previous model developed for SV in the adults and to account for irregular peaks (due to absorption) observed in the profiles [18]. One, two, and three compartment disposition models were investigated for SV. The structure of the final model for SV and the metabolites is shown in Fig. 1. The parameters for each of the analytes are as follows: SV—D_1_, F_1_, and ka_1_ are the duration of zero-order, fraction of the dose, and first-order rate constant, respectively, for the first absorption process. ALAG, D_2_, F_2_, and ka_2_ are the lag time, duration of zero-order, fraction of the dose, and the first-order rate constant, respectively, for the second absorption process. V_SL_ and CL_SLe_ are the volume of distribution and total elimination parameters. SVA—CL_LA_, V_SVA_, and CL_SVAe_ are the formation, volume of distribution, and total elimination parameters, respectively. HMSV—CL_LH_, V_HSV_, CL_Emax_, CL_EC50_, and γ are the formation, volume of distribution, maximum elimination, concentration of 50% metabolic rate, and sigmoidicity parameters, respectively. HMSVA—CL_AH_ and CL_SHVSHVA_, V_HSVA_, and CL_HSVAe_ are the formation from SVA, formation from HMSV, volume of distribution, and total elimination parameters, respectively. DHSV—CL_LD_, V_DHSV_, and CL_DHSe_ are the formation, volume of distribution, and total elimination parameters, respectively.

Since the volumes of distribution of the metabolites are not structurally identifiable, only apparent parameters were estimated; estimated parameters were the ratio of the formation or elimination parameters to the total volume of distribution of the metabolites. This approach allows correct interpretation of the effect of covariates on both formation/elimination and volume of distribution parameters during analysis. Random effect parameters that describe between-subject variability (BSV) in parameter estimates were modelled assuming exponential distribution; only variances of the variability were estimated and correlations between parameters led to convergence and model instability problems. Residual variability models were used to describe unexplained differences between log-transformed data for the analytes and log-transformed model predictions. The model used for the residual variabilities was the double exponential error model; this model is equivalent to the combined additive and proportional residual error model on the normal scale [5, 19]. For some analytes, a time-dependent model was used to describe their error model; different error terms were estimated for the absorption phase (0–2 h) and the elimination phase (2–8 h), detailed in Online Resources.

Due to a significant fraction of the data falling below the LLOQ (40% for SVA, Online Resource), it was not possible to treat these values as missing data and exclude the time points from the analysis. These data points were retained in the analysis but treated as censored data, and the likelihood of the model prediction to be indeed below LLOQ was maximised (M3 method) [19].

### The covariate model

The following covariates were explored for inclusion in the model: age, body weight, gender, height, lean body weight, body mass index, and six SNPs (three for *SLCO1B1* and three for CYP3A5). Initially, empirical Bayes estimates were used to screen covariates (in the presence of high shrinkage (> 40%), covariates were tested directly); for continuous covariates, a linear regression and for categorical covariates (including the SNPs), the ANOVA was performed to establish possible association between the covariates and the parameters. Identified covariates were incorporated in the model for evaluation using forward inclusion (change in objective function value, ΔOFV > 3.84, *p* ≤ 0.05, 1 degree of freedom) and backward elimination (ΔOFV > 7.88, *p* ≤ 0.005, 1 degree of freedom) procedures. The effect of body size (body weight and lean body weight) on formation/elimination and volume of distribution parameters was investigated using an allometric relationship centred on the median values, with fixed exponents of 0.75 and 1, respectively, or the exponents estimated from the data. Age was investigated as a covariate using relationships established by plotting age against empirical Bayes estimates for parameters. The effect of body size on parameters was retained in the final model only if its inclusion did not make the model worse, even if it did not improve the model based on ΔOFV. The effects of SNPs on parameters were investigated using fractional change relationships [5]. For each SNP, three different models were investigated: dominant, recessive, and additive genetic models. With the dominant model, individuals with two copies of the wild-type alleles were considered “typical” individuals and the reference group for the analysis, and individuals with at least one variant allele have a fractional change in parameter estimated from the data. For the recessive model, only individuals with two copies of the variant allele were considered different from the rest of the population, with fraction change in parameter estimated. Finally, for the additive model, individuals with one or two copies of the variant allele were considered distinct from each other, and the fractional change in parameter estimated for individuals with two variant copies is double the estimated fractional change for individuals with only one copy of the variant allele.

### Model evaluation

Structural and statistical components of the model were evaluated using GOF plots such as observation versus population (DV vs PRED) and individual prediction (DV vs IPRED), as well as conditional weighted residual versus population prediction (CWRES vs PRED) and time (CWRES vs TIME). Visual predictive check (VPC) was used to assess the predictive performance of the final model by simulating plasma concentration data for 1000 random individuals (for each dose) using covariate information from the original dataset. Combinations of covariates for individuals were sampled with replacement in order to retain the correct covariance structure of the covariates. A bootstrap (*n* = 500) analysis was performed to assess the robustness of the parameter estimates and to obtain the non-parametric confidence intervals on the final population PK parameter estimates.

## Results

In the current study, plasma concentrations of SV and its four metabolites from 32 children/adolescents were analysed together with demographic and genotype data. The body weights of individuals in this study were mostly outside of the expected range for the age group (Online Resource), in contrast to the heights. The population PK model that best described the plasma concentrations of SV, SVA, HMSV, HMSVA, and DHSV is presented in Fig. 1. Apparent parameters for the formation and elimination of SV and the metabolites were described by first-order processes using a one-compartment model. However, elimination of HMSV was described by a saturable non-linear process using the inhibitory sigmoid Emax model (Imax model). The parameter estimates (identifiable) for the final population PK model are presented in Table 1, together with the results of the bootstrap (95% non-parametric confidence intervals). BSVs were removed for some parameters where the estimates were close to the lower limit (zero) and caused instability during estimation.

**Table 1:** Parameter estimates of the final population PK model and bootstrap (95% non-parametric confidence interval) for SV, SVA, HMSV, HMSVA, and DHSV

Drug	Parameters	Structural model		Between subject variability (BSV^b^)	
		Estimate^a^	Bootstrap	Estimate [shrinkage(%)]	Bootstrap
SV	D_1_ (h^−1^)	0.069	0.064–0.073	–	–
	D_2_ (h^−1^)	0.39	0.34–0.42	2.07 [17]	1.75–2.27
	ka_1_ (h^−1^)	0.030	0.027–0.032	–	–
	ka_2_ (h^−1^)	0.41	0.38–0.46	0.41 [0.5]	0.38–0.45
	BA^*^	0.78	0.70 – 0.89	–	–
	ALAG (h)	0.18	0.17–0.21	1.70 [13]	1.40–2.09
	CL_SLe_/F (L/h)	1300	1210–1450	0.63 [3]	0.53–0.71
	V_SL_/F (L)	110	98.7–114	2.17 [27]	1.59–2.25
SVA	CL_LA_/V_SVA_ (h^−1^)	0.043	0.04–0.046	0.96 [9]	0.76–1.02
	CL_SVAe_/V_SVA_ (h^−1^)	0.13	0.11–0.14	0.79 [25]	0.60–0.81
	θ_561_: c.521T>C on V_SVA_	− 0.37	− 0.38 to − 0.35	–	–
	θ_562_: c.521T>C on CL_LA_	0.93	0.86–1.04	–	–
HMSV	CL_LH_/V_HSV_ (h^−1^)	16.1	15–21.5	0.26 [32]	0.23–0.28
	CL_Emax_/V_HSV_ (nM/h)	660	637–967	–	–
	CL_EC50_ (nM)	20	17.8–22.8	0.67 [6]	0.58–0.74
	γ	0.86	0.83–0.87	–	–
HMSVA	CL_AH_/V_HSVA_ (h^−1^)	0.48	0.39–0.5	0.33 [60]	0.27–0.36
	CL_HSVAe_/V_HSVA_ (h^−1^)	4.91	4.6–5.1	0.18 [47]	0.12–0.19
	CL_HSVHSVA_/V_HSVA_ (h^−1^)	0.55	0.52–0.62	0.51 [4]	0.45–0.56
	θ_AGE1_: age (Fra) on CL_HSVAe_	1 (fixed)	–	–	–
	θ_AGE2_: age (50%) on CL_HSVAe_ (year)	5.34	4.9–5.5	–	–
DHSV	CL_LD_/V_DHSV_ (h^−1^)	4.11	3.8–5.0	0.29 [34]	0.23–0.29
	CL_DHSe_/V_DHSV_ (h^−1^)	12.9	12.3–14.2	0.28 [42]	0.22–0.29
	θ_AGE3_: age (Fra) on CL_DHSe_	0.90	0.83–0.94	–	–
	θ_AGE4_: age (50%) on CL_DHSe_ (year)	6.3	5.9–6.6	–	–
Residual Variability^c^					
SV	eps1_SV_	–	–	0.38	0.33–0.44
	eps2_SV_			0.32	0.28–0.35
	eps3_SV_			0.30	0.29–0.33
	m_SV_			0.012	0.011–0.012
SVA	eps1_SVA_			0.26	0.23–0.29
	eps2_SVA_			0.16	0.13–0.16
	m_SVA_			0.22	0.19–0.24
HMSV	eps1_HMSV_			0.29	0.27–0.34
	eps2_HMSV_			0.24	0.22–0.28
	eps3_HMSV_			0.12	0.11–0.28
	m_HMSV_			0.0001 (fixed)	–
HMSVA	eps1_HMSVA_			0.18	0.16–0.20
	eps2_HMSVA_			0.1	0.095–0.11
	m_HMSVA_			0.0001 (fixed)	–
DHSV	eps1_DHSV_			0.24	0.21–0.27
	eps2 _DHSV_			0.21	0.19–0.23
	eps3 _DHSV_			0.086	0.08–0.09
	m_DHSV_			0.0001 (fixed)	–

*F_1_ = 1/(1 + BA), *F*_2_ = BA/(1 + BA)

^a^Population parameter estimates for a typical individual in the study, 14 years old, 80 kg and homozygous variant CC genotype for the rs4149056

^b^BSV is expressed as CV (coefficient of variation) calculated as $\sqrt{\left({e}^{\omega^2}-1\right)}$

^c^Residual error variability for the analytes were based on the double exponential error model for log-transformed data with time dependency for some analytes. ln(*y*) = ln(*f* + *m*) + (*f*/(f + *m*)ε_1_ + (*m*/(*m* + *f*))ε_2_ where *y* is the observed concentration, *f* is the model prediction, *m* is a positively constrained parameter, and ε_1_ and ε_2_ are random errors, assumed to be normally distributed with means of zero and variance of eps1 and eps2, respectively. *m* is estimated or fixed to an estimate around or lower than the LLOQ in order to minimise bias

SV—eps1_SV_ and eps2_SV_ correspond to 0–2 h and 2–8 h for ε_1_ and eps3_SV_ and m_SV_ to ε_2_ and *m*, respectively. SVA—eps1_SVA_, eps2_SVA_, and m_SVA_ correspond to ε_1_, ε_2_, and *m* respectively. HMSV—eps1_HMSV_ and eps2_HMSV_ correspond to 0–2 h and 2–8 h for ε_1_ and eps3_HMSV_ and m_HMSV_ to ε_2_ and *m*, respectively. HMSVA—eps1_HMSVA_, eps2_HMSVA_, and m_HMSVA_ correspond to ε_1_, ε_2_, and *m*, respectively. DHSV—eps1_DHSV_ and eps2_DHSV_ correspond to 0–2 h and 2–8 h for ε_1_ and eps3_DHSV_ and m_DHSV_ to ε_2_ and *m*, respectively

The unexplained variability was described by a double exponential error model with time dependency for SV, HMSV, and DHSV (Table 1). Table 2 illustrates the important steps in the covariate model development, including ΔOFV during the backward deletion process and the associated *p* values for the covariates. The combined effect of the covariates improved the model fitting substantially, as the total ΔOFV was 267.58 compared with the base model. The final population PK model included the additive effect of c.521T>C on CL_LA_/V_SVA_ (both on CL_LA_ and V_SVA_), age on CL_HSVAe_ and CL_DHSe_; the covariate relationships are described by Eqs. 1–4:

1 $$ \frac{C{L}_{LA}}{V_{SVA}}=0.043\cdot \frac{\left(1+ rs56\cdot 0.93\right)}{\left(1+ rs56\cdot -0.37\right)} $$

2 $$ \frac{C{L}_{SVA e}}{V_{SVA}}=0.13\cdot \frac{1}{1+ rs56\cdot -0.37} $$

3 $$ \frac{C{L}_{HSVA e}}{V_{HSVA}}=4.91\cdot \left(1-\frac{1\cdot \mathrm{Age}}{5.34+\mathrm{Age}}\right) $$

4 $$ \frac{C{L}_{DHSe}}{V_{DHSV}}=12.9\cdot \left(1-\frac{0.90\cdot \mathrm{Age}}{6.3+\mathrm{Age}}\right) $$

where rs56 is a dummy variable that takes the value of 0, 1, and 2 for individuals that belong to the wild type (TT), heterozygous variant (TC), and homozygous variant (CC) genotypes for *SLCO1B1* c.521T>C, respectively.

**Table 2:** Summary of important steps in the SV metabolite covariate model development

Model	OFV	ΔOFV	*df*	*p* value
Final model	− 802.89	–	–	–
Age on CL_DHSe_	− 701.39	101.50	2	< 0.001
Age on CL_HSVAe_	− 674.56	127.33	2	< 0.001
c.521T>C on CL_LA_	− 785.04	17.85	1	< 0.001
c.521T>C on V_SVA_	− 781.99	20.90	1	< 0.001

**Fig. 2:**
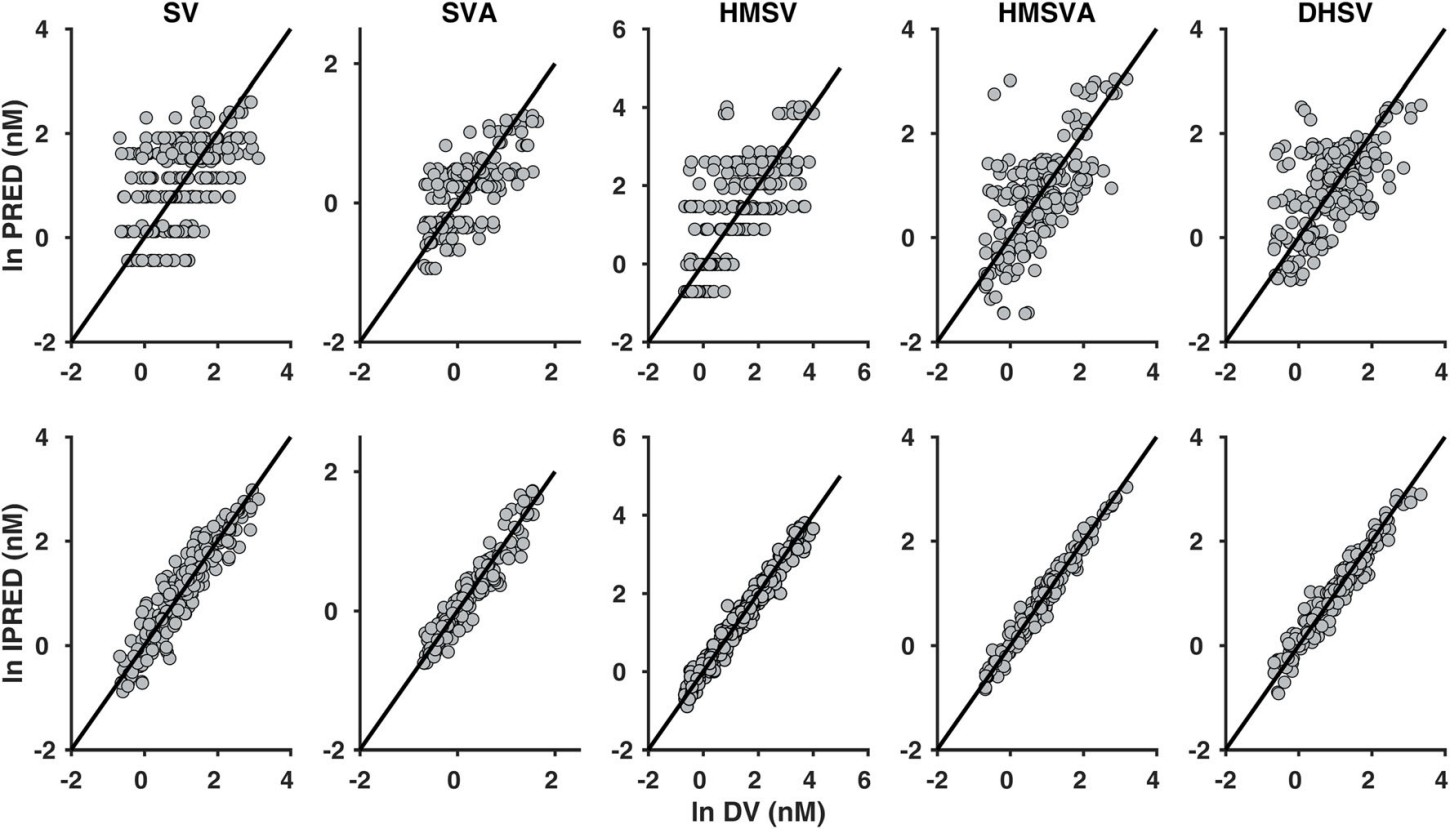
Goodness-of-fit (GOF) plots for the final population PK model. Population prediction (PRED) vs observed data (DV) and individual prediction (IPRED) vs observed data (DV) for SV, SVA, HMSV, HMSVA, and DHSV. The dark continuous lines are the line of unity

**Fig. 3:**
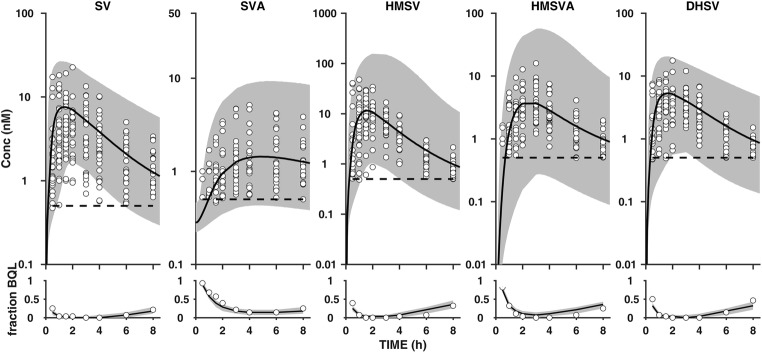
Visual predictive check (VPC) of the final model following a 10-mg oral dose of SV. In the upper panels, open circles represent the observed plasma concentration data, the grey areas are the areas between 5th and 95th percentiles, the dark solid lines are the 50th percentiles, and the horizontal dark dashed lines are the LLOQ for the analytes. In the lower panels, the open circles represent the observed fraction of samples below LLOQ, the grey areas are the simulated 90% confidence intervals of the fraction below LLOQ, and the solid dark lines are the simulated median fraction below LLOQ at each time points for the analytes

**Fig. 4:**
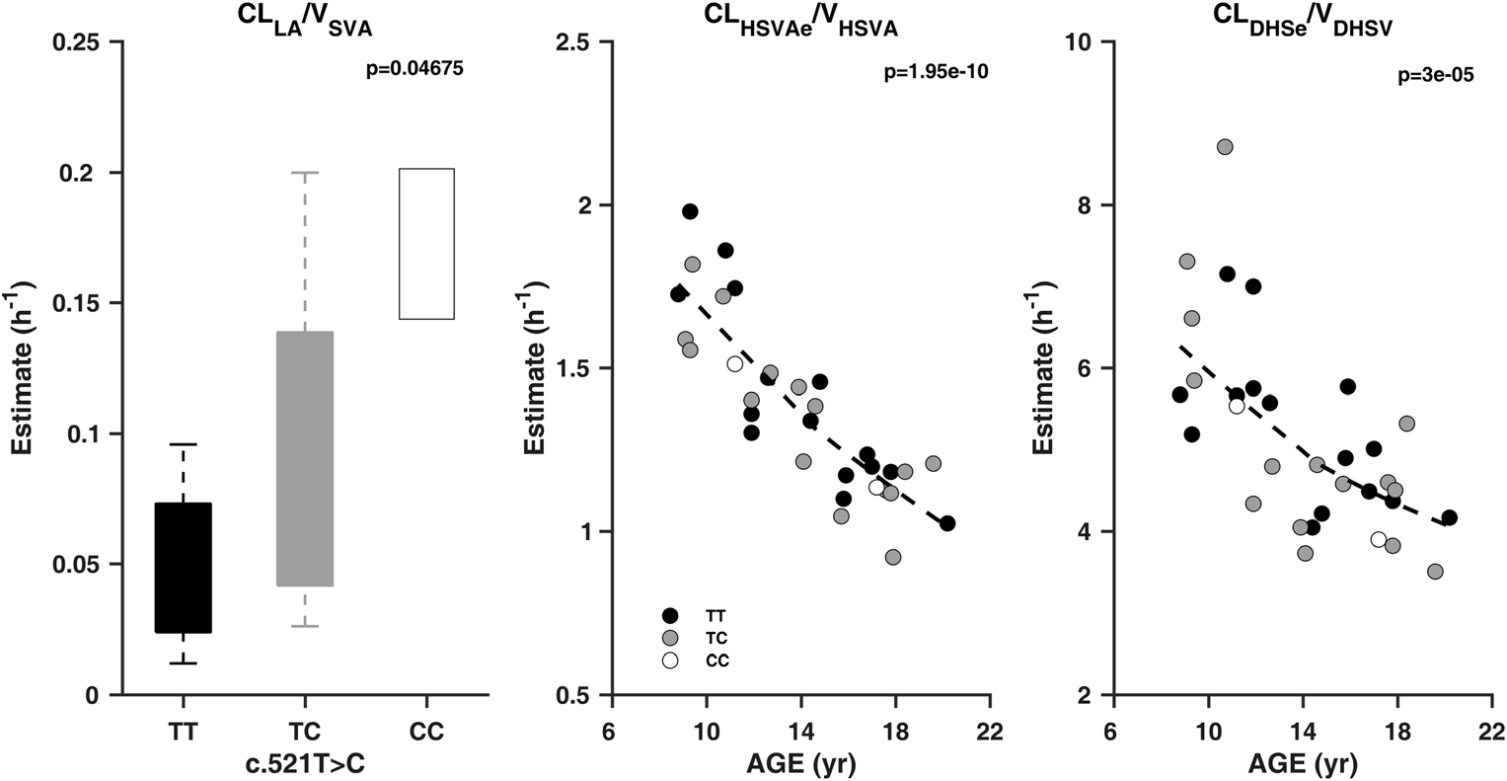
Empirical Bayes estimates versus covariates, showing covariate effects in the final population PK model; CL_LA_/V_SVA_ versus c.521T>C, CL_HSVAe_/V_HSVA_ versus age, and CL_DHSe_/V_DHSV_ versus age. For continuous covariate (age), the dark dashed line is a locally weighted scatter plot smooth (lowest) line for the data

The GOF plots of the final model are shown in Fig. 2**,** with population and individual predictions plotted against observed plasma concentrations. The VPC (1000 simulations) for individuals that received 10 mg in the original dataset is shown in Fig. 3. These plots also include the fraction of samples below LLOQ at each time point for SV and the metabolites. The VPC for 10-mg dose was also stratified by c.521T>C genotype for comparison of observed plasma concentrations and simulated median profile for each of the genotypes (Online Resource). Other GOF plots, CWRES vs PRED and CWRES vs TIME, are presented in Online Resource, together with the VPC for 20-mg dose and individual plasma concentration-time data with the fitted profiles (population and individual fits). These plots showed that the developed population model for SV and the metabolites adequately describes the observed plasma concentrations in children and adolescents both in terms of central tendency and variability in the data, as well as fractions below the LLOQ. However, there is slight over-prediction of variability for HMSV and HMSVA. Figure 4 illustrates empirical Bayes estimates of parameters that have been significantly influenced by covariates in the final model plotted against the covariates (CL_LA_/V_SVA_ vs rs41496056, CL_HSVAe_/V_HSVA_ vs age, and CL_DHSe_/V_DHSV_ vs age).

Based on the adult SV-SVA population PK model [5], the effect of c.521T>C genotype was explored on the parameters associated with both the formation and the elimination of SVA, namely CL_LA_, V_SVA_, and CL_SVAe_. Since only apparent parameters are estimated in the model, the effect of c.521T>C can only be incorporated separately on two of the parameters. In this analysis, the most significant combination was CL_LA_ and V_SVA_; the removal of the covariate led to ΔOFV (increase) of 38.75 (*p* value < 0.001, 2 degrees of freedom). This significant and pronounced effect of the covariate on plasma concentrations of SVA is also illustrated in the VPC plots (Online Resource), where observed and simulated median plasma concentration profiles resulted in up to 6.3-fold higher exposure in c.521CC subjects compared with that in c.521TT individuals.

The final model also included a significant effect of age on CL_HSVAe_ and CL_DHSe_ elimination parameters for the HMSVA and DHSV metabolites. The removal of this covariate led to substantial ΔOFV (increase) of 127.33 and 101.50 for CL_HSVAe_ (*p* value < 0.001, 2 degrees of freedom) and CL_DHSe_ (*p* value < 0.001, 2 degrees of freedom), respectively. The covariate model suggests that for both HMSVA and DHSV, elimination clearance parameters decreased with age: 60% and 49% as age increased from 4 to 18 years for CL_HSVAe_ and CL_DHSe_, respectively. These reductions followed a sigmoid Imax function with the fraction of maximum reduction possible fixed at 1 for CL_HSVAe_ and estimated to be 0.90 for CL_DHSe_; a 50% decrease occurred at 5.3 years for CL_HSVAe_ and 6.3 years for CL_DHSe_.

## Discussion

The population PK model of SV and its metabolites in children and adolescents identified genetic polymorphism in *SLCO1B1* c.521T>C as an important covariate on SVA systemic exposure, consistent with the results obtained in adults. The model successfully captured the more pronounced effect of this genotype in children and large variability in the dose-exposure relationship within *SLCO1B1* c.521T>C genotype groups. The SV metabolite model has provided further understanding of possible metabolic elimination of SV and its metabolites in children, especially the formation of HMSVA from SVA which may contribute to low/undetectable plasma concentrations of SVA in some individuals. Such extensive clinical data for multiple SV metabolites or modelling efforts have not been reported in the adults.

In contrast to adult SV models, a one-compartment model was used to describe the PK of SV in children/adolescents due to sampling restricted to 8 h in this study, as opposed to 24–36 h in the adult studies; this may also have implications for parameter estimates obtained from this analysis [5, 15, 18]. The effect of sampling time is evident in the median plasma concentration-time profile for SV where a significant amount of the drug is still in the body when sampling stopped at 8 h (Fig. 3), making estimation of a second compartment from the data challenging. A one-compartment disposition model and first-order rates were also used to describe the PK of the metabolites, except for the elimination of HMSV. The latter was described by a saturable process, possibly due to either potential saturation of elimination or formation of the metabolites which is being estimated through the elimination of the metabolite as the rate-limiting step.

The model incorporated the formation of HMSVA from both SVA and HMSV via CYP3A4/5 metabolism and hydrolysis, respectively, based on in vitro evidence in adults [6]. These multiple routes of HMSVA formation implemented in the model allowed adequate prediction of SVA concentrations. The assumption that HMSVA is not formed solely from SVA was also reinforced by the fact that the time at which maximum concentration (Tmax) was observed for this metabolite was earlier than SVA Tmax, but later than HMSV Tmax. Although the formation of the acid metabolites is not a one-way process, back conversion via acyl-glucuronide was not considered due to model identifiability problems. In addition, back conversion of SVA to SV had no significant improvement on objective function and diagnostic plots in the model developed from adult studies [5].

In addition to V_SVA_, the model indicated that c.521T>C significantly affects SVA formation parameter (CL_LA_) rather than CL_SVAe_, as reported in adult population PK model [5]. This result is unexpected since SV uptake into the liver is a predominantly passive process and the formation of SVA from SV in the liver is therefore not expected to be affected by c.521T>C genotype. This outcome of the modelling could be due to the limitations imposed by the shorter sampling duration in this study, thereby making it difficult to introduce covariates on the parameters and estimate CL_SVAe_ accurately.

The effect of age on HMSVA and DHSV elimination clearances (CL_HSVAe_ and CL_DHSe_) is another important finding of the current work; these present additional sources of variability in SVA systemic exposure to c.521T>C in hypercholesterolemic children and adolescents. Body weight and lean body weight were both found to have no significant effect on any model parameters both with the exponents on CL and V fixed or estimated; in fact, they made the model worse by increasing the objective function when included. It was to some extent unexpected to see a reduction in the parameter values (absolute) with increasing age, since enzyme maturation and renal function are generally expected to increase with age, reaching the adult level at some point during development. The children in this study were between 8 and 20 years old; therefore, clearance was expected to be fairly constant or increase with age. For most formation and elimination parameters, this was the case, with the exception of CL_HSVAe_ and CL_DHSe_. The fact that most children in this study were overweight (Online Resource) may rationalise unexpected trends observed with age, as overweight and obese children and adolescents have been reported to have higher absolute clearance for midazolam and metformin compared with adults with normal body weight and obese adults [20, 21].

Out of the six SNPs investigated in this work, only c.521T>C showed a significant effect on the SVA plasma concentration, analogous to the effect on SVA plasma concentrations in adults [5]. None of the other SNPs for either OATP1B1 or CYP3A5 showed a significant effect on the PK of SV or any of its metabolites. One of the SNPs analysed in this study (CYP3A5*3 – rs776746) was reported to be a significant covariate for SV-SVA conversion in adults [5]; however, this was not the case here. This finding may be attributed to the sample size and the proportion of subjects in different CYP3A5 genotype groups (zero, four, and 28 individuals with AA, AG, and GG, respectively; Online Resource) and therefore the lack of power to identify the effect of this SNP. During the initial screening of the covariates with the empirical Bayes estimates of the basic model, CYP3A5*3 showed a marginally significant effect on the conversion of SV to DHSV (formation parameter for DHSV); however, this effect was not evident once age was considered as a covariate. Age, body weight, and lean body weight also showed initial significant effects (marginal) on absorption parameters such as D_2_, ka_2_, and ALAG; however, these effects were not apparent once other covariates (e.g. c.521T>C and age) were introduced in the model. In contrast to the adult population model [5], age was not a significant covariate for SV bioavailability, which may be due to the age range of this cohort and the sample size. Ethnicity was another important covariate incorporated in the adult SV-SVA population PK model [5]; Japanese and other Asians have been reported to be associated with higher clearance of SV and therefore higher exposure of SVA compared with other individuals but this could not be investigated here as majority (*n* = 30) of the children and adolescents were of the Caucasian descent.

The population PK model developed in this study incorporates also information from data below LLOQ which is higher compared with most adult studies (0.25 nM) [5]. The use of the M3 method to maximise the likelihood of the model to predict plasma concentration below LLOQ allowed the model to adequately describe the data (Fig. 3).

The population PK model developed in this work is adequate for the intended purpose, i.e. to investigate the effect of common SNPs and demographic covariates on the PK of SV and four metabolites in children and adolescents for possible dose optimisation. This model is entirely data-driven and based on a compartmental model framework; the empirical nature of this approach can be considered a limitation. The short duration of sampling for this study which has implication for parameters estimated in the current analysis, also means extrapolated of this model beyond concentration range observed in the current analysis, has to be done with caution. Important mechanistic information such as pre-systemic formation of the metabolites as well as drug concentrations at the site of efficacy (liver) and toxicity (muscle) could not be adequately accounted for. A model with a physiologically based structure is required to predict SVA tissue exposure and assess consequences of the variability on the pharmacodynamics of this drug in the children/adolescents.

In summary, this study reports quantitatively the significant effect of *SLCO1B1* c.521T>C and age on the PK of SV and its metabolites in children and adolescents. The population model described inter-individual variability in the exposure of parent drug and all metabolites in the children. In particular, the model captured cohort of the paediatric participants (25%) where SVA systemic exposure was negligible. It is envisaged that the model can be applied for Monte Carlo simulations using identified covariate relationships to match plasma concentrations that have been linked to efficacy in adults. Despite a relatively small sample size, the modelling work presented here represents an important step towards optimisation of SV dosing in the children and adolescents.

## Electronic supplementary material

ESM 1 (PDF 1315 kb)
