# Supplementary material for: A population pharmacokinetic model for simvastatin and its metabolites in children and adolescents
Source: Eur J Clin Pharmacol. 2019 Jun 6;75(9):1227–35. doi: 10.1007/s00228-019-02697-y (PMC6697721; doi:10.1007/s00228-019-02697-y)
Supplement: Supplementary file 1 — (PDF 1315 kb) [file 228_2019_2697_MOESM1_ESM.pdf]

Supplementary Material - A population pharmacokinetic model for simvastatin and its metabolites in children and adolescents

Kayode Ogungbenro, PhD[1], Jonathan B. Wagner, DO[2-4], Susan Abdel-Rahman, PharmD[3,4], J. Steven Leeder, PhD[3,4] and Aleksandra Galetin, PhD[1]

[1]Centre for Applied Pharmacokinetic Research,

Division of Pharmacy and Optometry,

School of Health Sciences,

Faculty of Biology, Medicine and Health,

University of Manchester, Manchester Academic Health Science Centre,

Manchester, M13 9PT, United Kingdom

[2]Ward Family Heart Center and [3]Division of Clinical Pharmacology, Toxicology and Therapeutic Innovation, Children's Mercy Kansas City, Kansas City, MO; [4]Department of Pediatrics, University of Missouri-Kansas City School of Medicine, Kansas City, MO

Supplementary Method

The residual unexplained variability in the data was modelled using a double exponential error model. This error model was proposed for log-transformed data by Beal [1] and it is equivalent to combined additive and proportional residual error model for un-transformed data. To minimise bias in the model prediction, a parameter *m* is added which can be fixed or estimated to a value that is lower than the lower limit of quantification. Also, time-dependent variances were estimated for the unexplained residual errors for SV, HMSV and DHSV. This was used to estimate different error terms for the ascending (absorption) and descending (elimination) phases of the profile. This is particularly important for plasma concentration profiles that is characterised by erratic absorption. This approach was proposed by Karlsson et al [2] and was used to describe the PK profile of SV and SVA in adults [3].

Supplementary Results

The results of the covariate analysis showed that that individuals carrying one or two variant alleles for this SNP (c.521TC and CC, respectively) have 37% (95% CI: 35 – 38%) and 74% (95% CI: 70 – 76%) lower VSVA compared to homozygous wild-type individuals (c.521TT). In addition, c.521TC and CC individuals have 93% (95% CI: 86 – 104%) and 186% (95% CI: 172 – 208%) higher CLLA values, respectively compared to the c.521TT individuals. The combined effect of c.521T>C resulted in 206% (95% CI: 200 – 214%) and 1000% (95% CI: 927 – 1033%) higher ratio of CLLA/VSVA in c.521TC and CC subjects, respectively compared to c.521TT subjects. Inclusion of this covariate in the model significantly improved the fitting, especially to SVA plasma concentrations.

Despite the use of complex absorption model to describe the absorption of SV using two parallel absorption processes based on a previous model developed for SV in the adults and

to account for irregular peaks there is slight under prediction at the initial phase of the profile. This is probably due limited sampling during the absorption phase for SV with a very rapid absorption rate.

In Table 1 is the shrinkage associate with the empirical Bayesian estimates, high shrinkage (>40%) were associated with CL_{AH}/V_{HSVA} (60%), CL_{HSVAe}/V_{HSVA} (47%) and CL_{DHSe}/V_{DHSV} (42%) and for these parameters covariates were tested directly using likelihood ratio test. For most parameters the shrinkage calculated was low or moderate.

The PK of HMSV metabolites was better described by a saturable process compared to a first order process. This need to be interpreted with caution given the short duration (0 – 8 h) of sampling for this study, and therefore has implication for reliability of the parameter estimates. Also extrapolation of this model beyond the concentration range observed in the current analysis has to be done with caution given the empirical nature of the model used for the analysis.

The VPC for 10mg (Fig 3) and 20mg (Supplemental Figure S3) doses showed adequate prediction of the central tendency in the data and variability for SV, SVA and DHSV. However there is over-prediction of the variability for HMSV and HMSVA metabolites. This could be due to the short duration (0 – 8 h) of sampling for this study; which is particularly important for 20mg dose.

Supplementary Discussion

This model incorporates information from data below LLOQ which is higher compared to adult studies. For SV and other metabolites a significant amount of the data was below LLOQ especially before 1 h and after 7 h (40% for SVA). Overall, the fractions of the data below LLOQ at different time points were described appropriately by the model both at the beginning and at the end of the profiles, in particular for SVA. The model also adequately

described plasma concentration data when stratified by the rs4149056 genotypes (Supplemental Figure S3) and captured fractions of SVA data below LLOQ in different SLCO1B1 c.521T>C genotype groups (very high for c.521TT subjects at all time points compared to CC individuals).

Supplemental Table S1: Demographics of the participants, the number of individuals in different genotypes of the measured SNPs and the total number of samples of the analyte used for the analysis.

Characteristics		Dose group 10mg	20mg	All
Demographics	Sample Size	28	4	32
	Age (yr)	13.4 (8.8 – 17.9)	19 (17.8 – 20.2)	14.1 (8.8 – 20.2)
	Body Weight (kg)	74.8 (30.2 – 138)	111.3 (65.6 – 149.2)	79.4 (30.2 – 149.2)
	Lean Body Weight (kg)	39.4 (9.6 – 75.8)	48 (19.4 – 63.9)	40.5 (9.6 – 75.8)
	Height (cm)	159.8 (133.2 – 181.9)	163.5 (159.6 – 168.3)	160.2 (133.2 – 181.9)
	Body Mass Index (kg/m^2)	28.6 (15.08 – 53.04)	41.6 (24.5 – 52.7)	30.2 (15.1 – 53.0)
	Gender (M/F)[§]	15/13	0/4	15/17
SLCO1B1 c.521T>C	rs72559745 (GG/AG/AA)	26/2/0	4/0/0	30/2/0
	rs4149056 (TT/TC/CC)	13/13/2	2/2/0	15/15/2
	rs2306283 (AA/AG/GG)	9/13/6	1/3/0	10/16/6
CYP3A5	rs15524 (TT/TC/CC)	27/1/0	3/1/0	30/2/0
	rs776746 (AA/AG/GG)	0/3/25	0/1/3	0/4/28
	rs10264272 (CC/TC/TT)	28/0/0	4/0/0	32/0/0
Number of Samples	SV	206	32	238 (7%)[§§]
	SVA	131	29	160 (38%)[§§]
	HMSV	199	31	230 (10%)[§§]
	HMSVA	179	28	207 (19%)[§§]
	DHSV	190	31	221 (14%)[§§]

For demographics the numbers are the mean and the range (minimum and maximum), [§]number of subjects that are male and female (M/F), [§§]percentage of the total number of samples below the lower limit of quantification (LLOQ).

Supplemental Figure S1 Plots of simulated body weight (WT) and height (HT) versus Age for 1000 children, adolescents and adults (1 and 25 yrs) with ideal body weight and height, superimposed with the observed values of body weight and height in the current dataset.

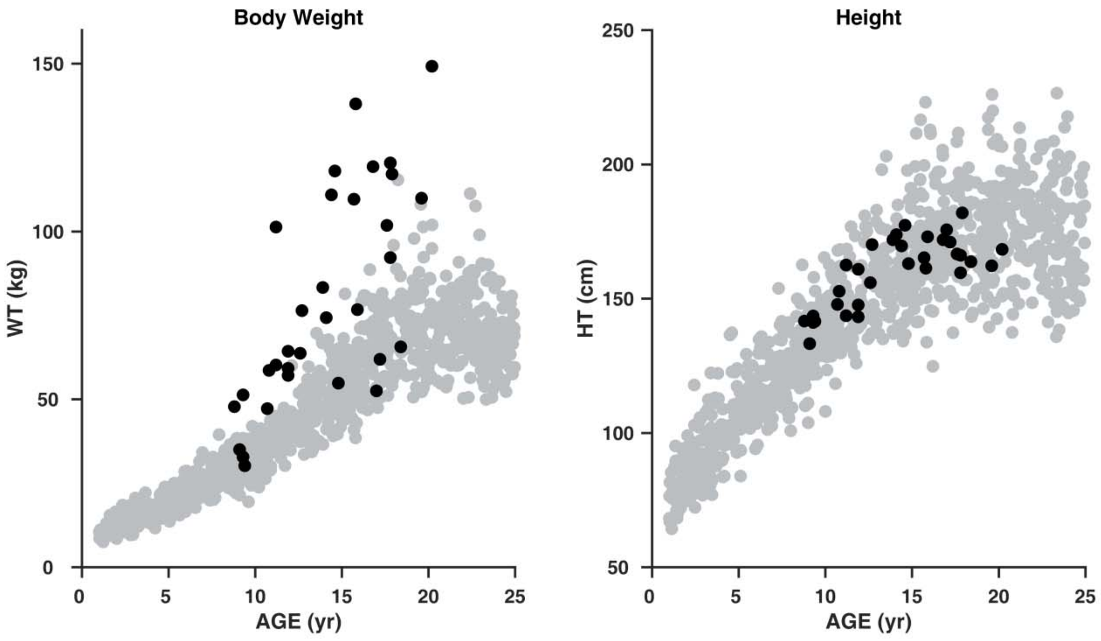

Supplemental Figure S2 Visual predictive check (VPC) of the final model following 20mg oral dose of SV. In the upper panels, open circles represent the observed plasma concentration data, the grey areas are the areas between 5th and 95th percentiles, the dark solid lines are the 50th percentiles and the horizontal dark dashed lines are the lower LLOQ for the analytes. In the lower panels, the open circles represent the observed fraction of samples below LLOQ, the grey areas are the simulated 90% confidence intervals of the fraction below LLOQ and the solid dark lines are the simulated median fraction below LLOQ at each time points for the analytes.

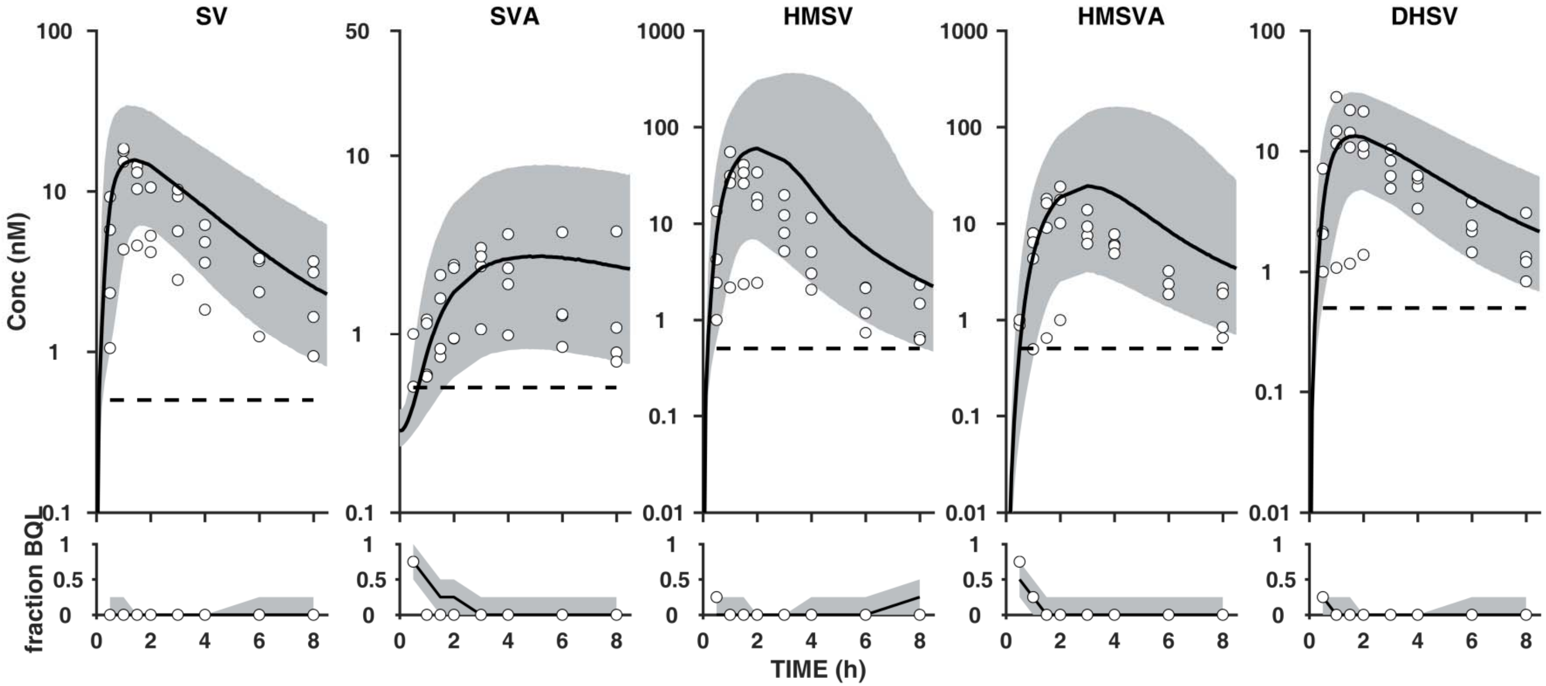

Supplemental Figure S3 Visual predictive check stratified by c.521T>C genotypes of the final model following 10mg oral dose of SV. In the upper panels the dark, grey and white circles are the observed plasma concentration data for TT, TC and CC groups, respectively and the continuous dark, dashed grey and dotted lines are the median predictions (50th percentiles) for TT, TC and CC groups, respectively. In the lower panels, the grey continuous, dashed and dotted lines are the observed fraction of the data below LLOQ for TT, TC and CC groups, respectively and the dark continuous, dashed and dotted dark lines are the median simulated fraction of the data below LLOQ for c.521TT, TC and CC groups, respectively.

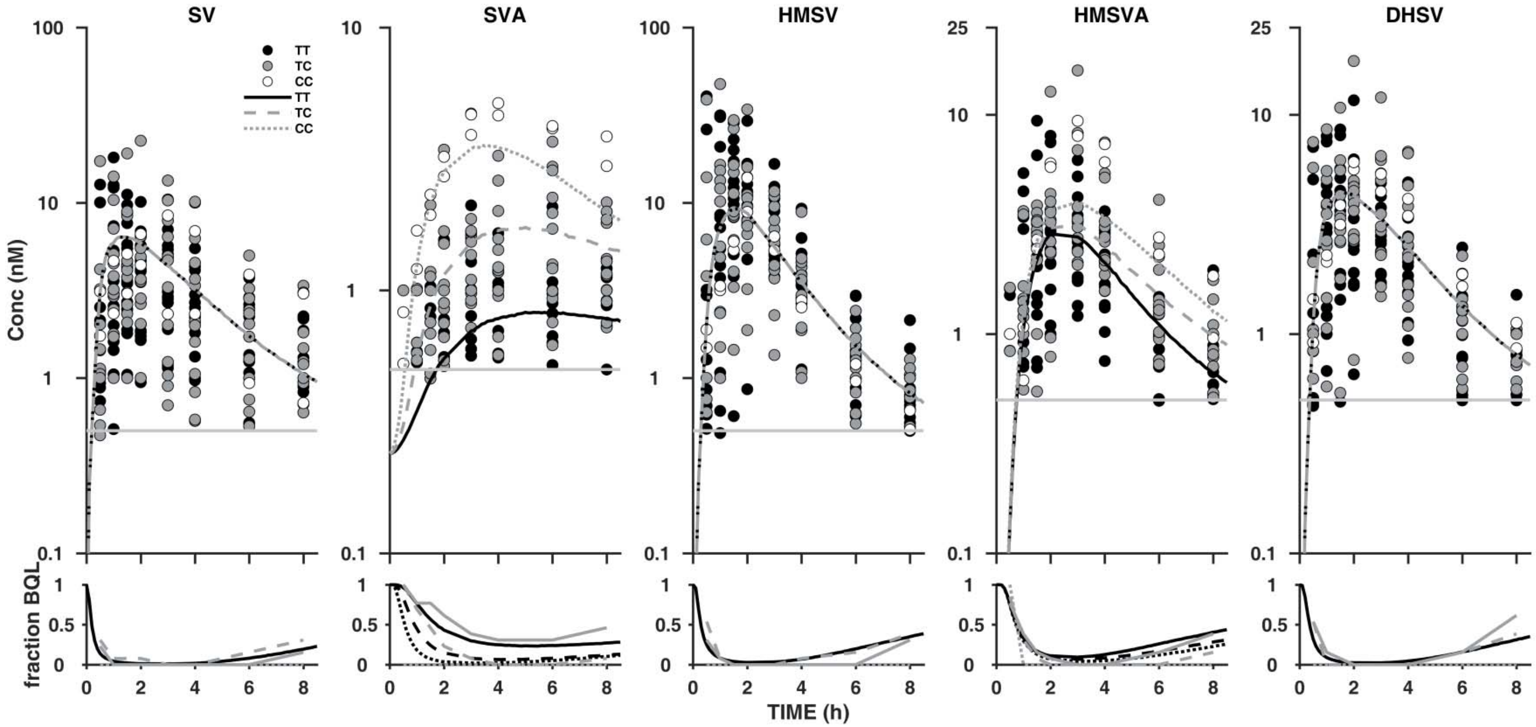

Supplemental Figure S4 Final model Goodness-of-fit (GOF) plots of conditionally weighted residual (CWRES) versus population prediction (PRED) and Time for SV, SVA, HMSV, HMSVA and DHSV.

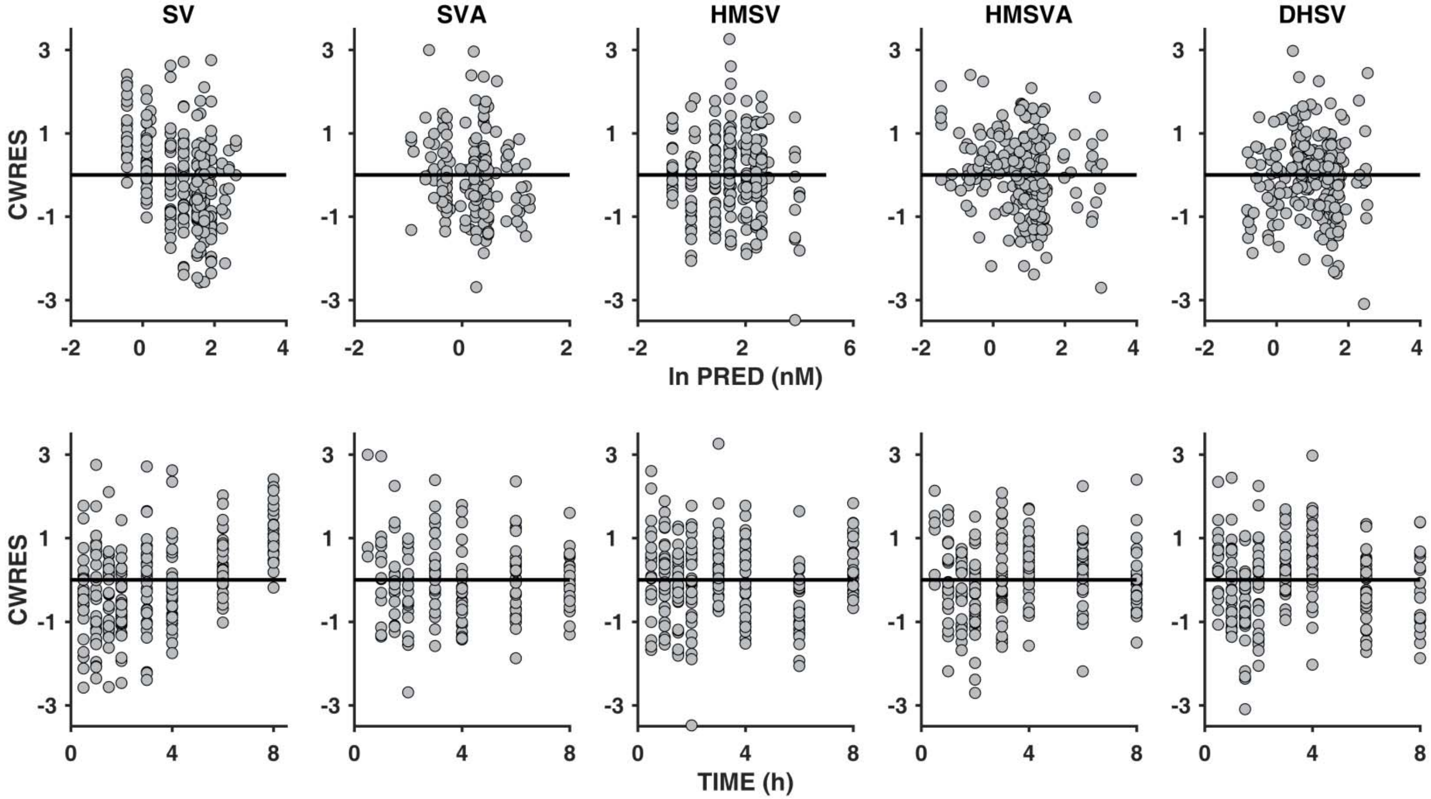

Supplemental Figure S5 Plots of SV plasma concentration data and fitted profiles for individuals after 10 and 20 mg oral dose of SV. The grey circles are the observed plasma concentration data, the dark continuous lines are the population model predictions and the dark dashed lines are the individual model predictions.

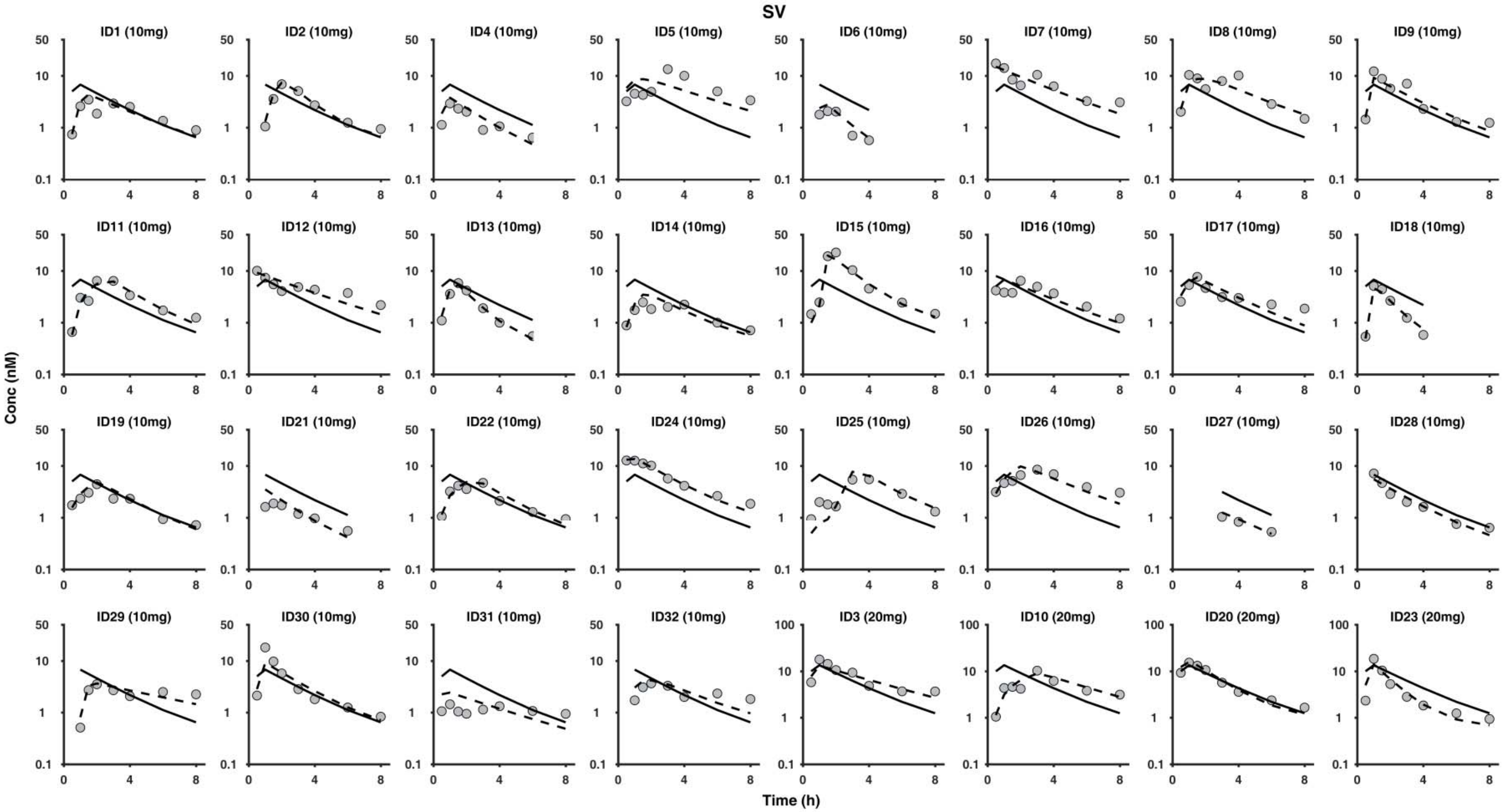

Supplemental Figure S6 Plots of SVA plasma concentration data and fitted profiles for individuals after 10 and 20 mg oral dose of SV. The grey circles are the observed plasma concentration data, the dark continuous lines are the population model predictions and the dark dashed lines are the individual model predictions.

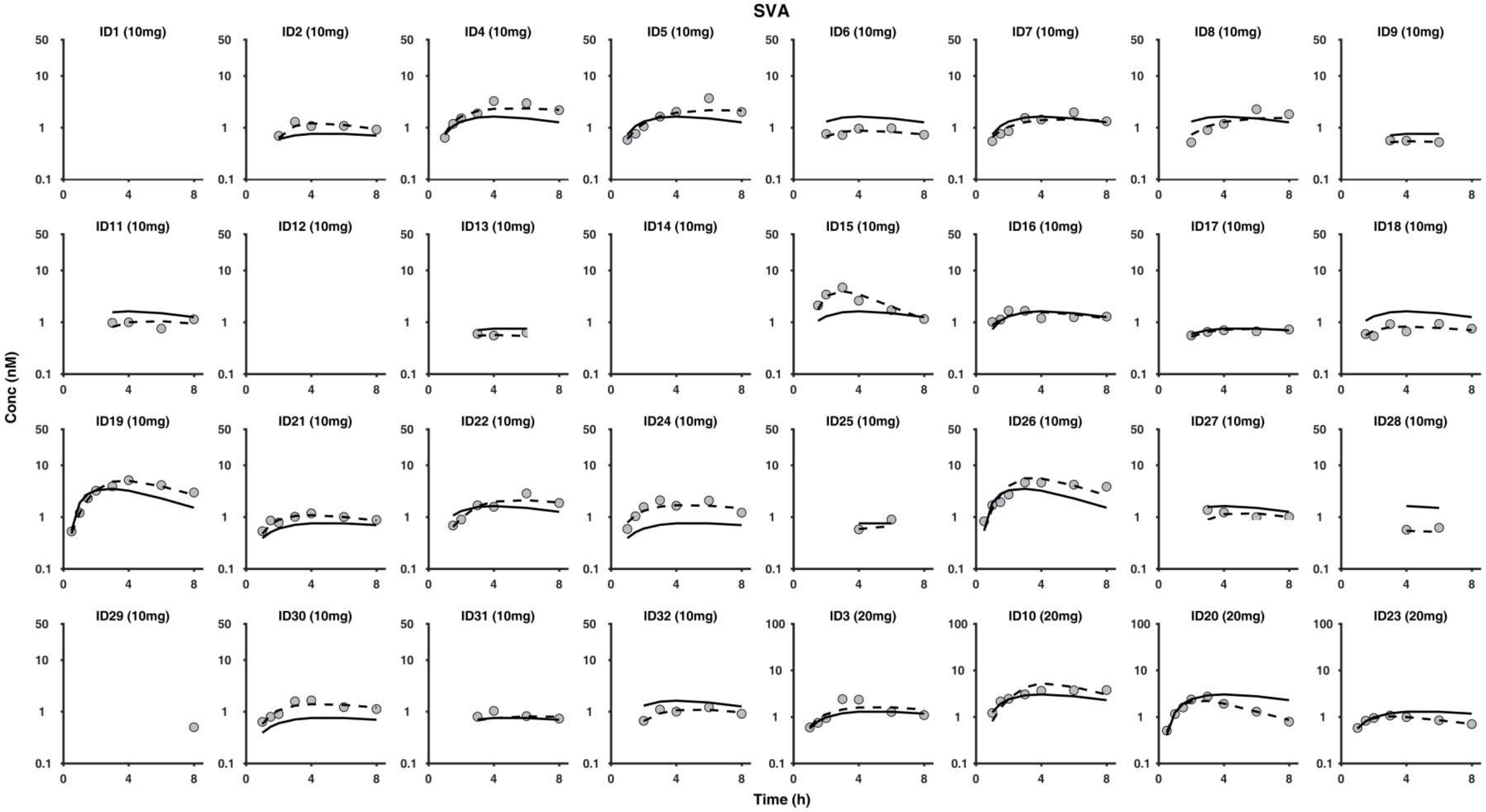

Supplemental Figure S7 Plots of HMSV plasma concentration data and fitted profiles for individuals after 10 and 20 mg oral dose of SV. The grey circles are the observed plasma concentration data, the dark continuous lines are the population model predictions and the dark dashed lines are the individual model predictions.

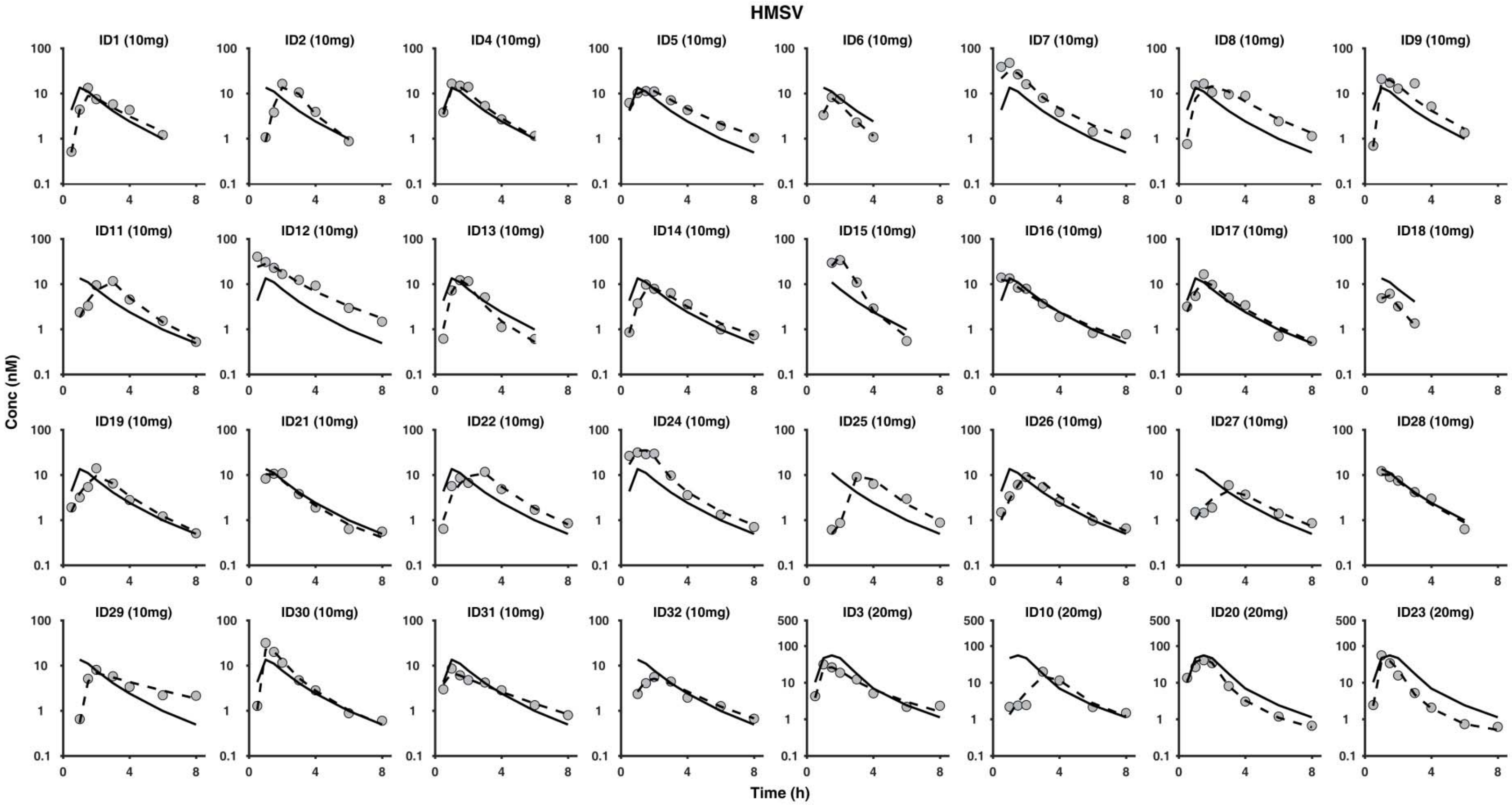

Supplemental Figure S8 Plots of HMSVA plasma concentration data and fitted profiles for individuals after 10 and 20 mg oral dose of SV. The grey circles are the observed plasma concentration data, the dark continuous lines are the population model predictions and the dark dashed lines are the individual model predictions.

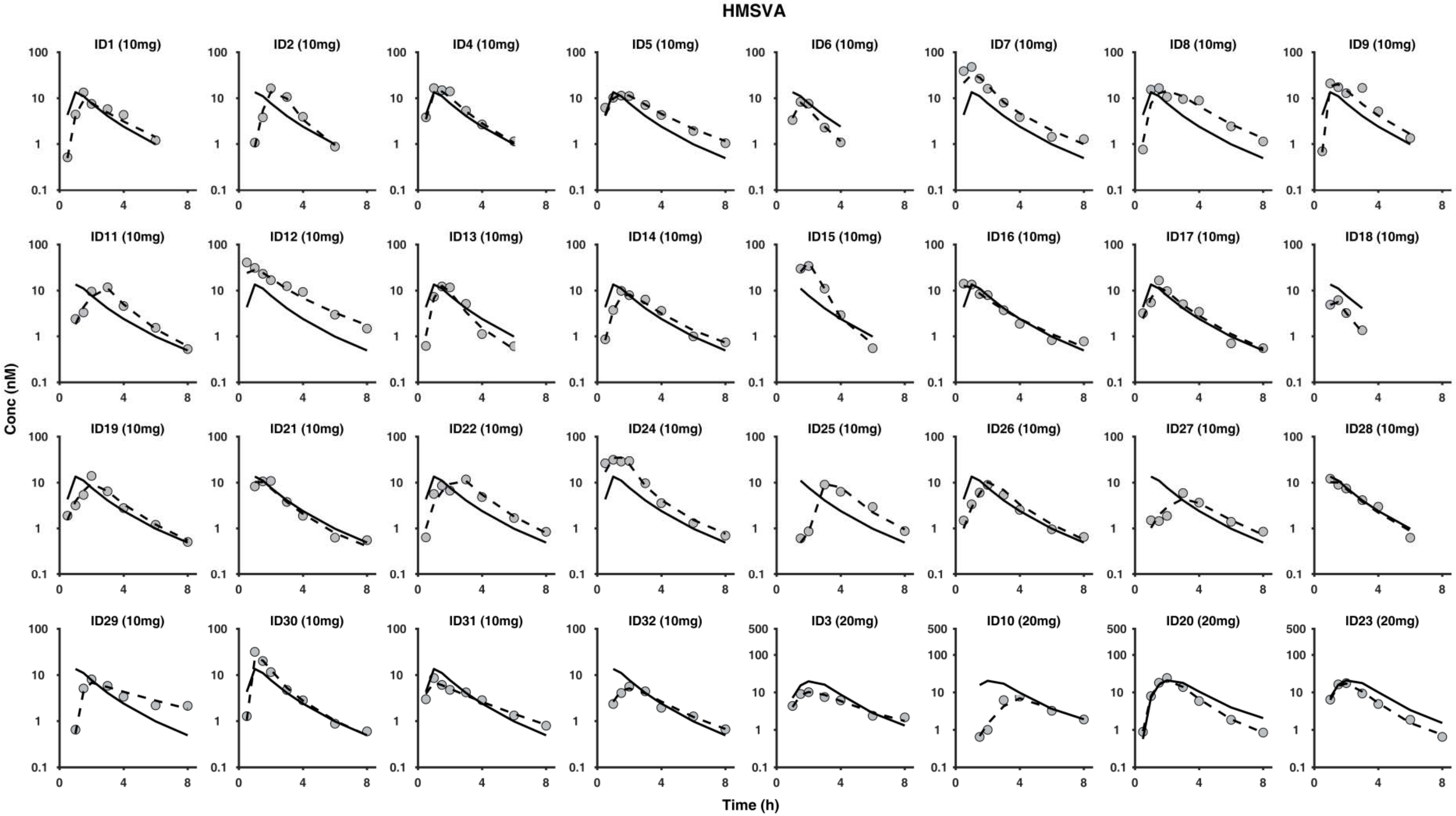

Supplemental Figure S9 Plots of DHSV plasma concentration data and fitted profiles for individuals after 10 and 20 mg oral dose of SV. The grey circles are the observed plasma concentration data, the dark continuous lines are the population model predictions and the dark dashed lines are the individual model predictions.

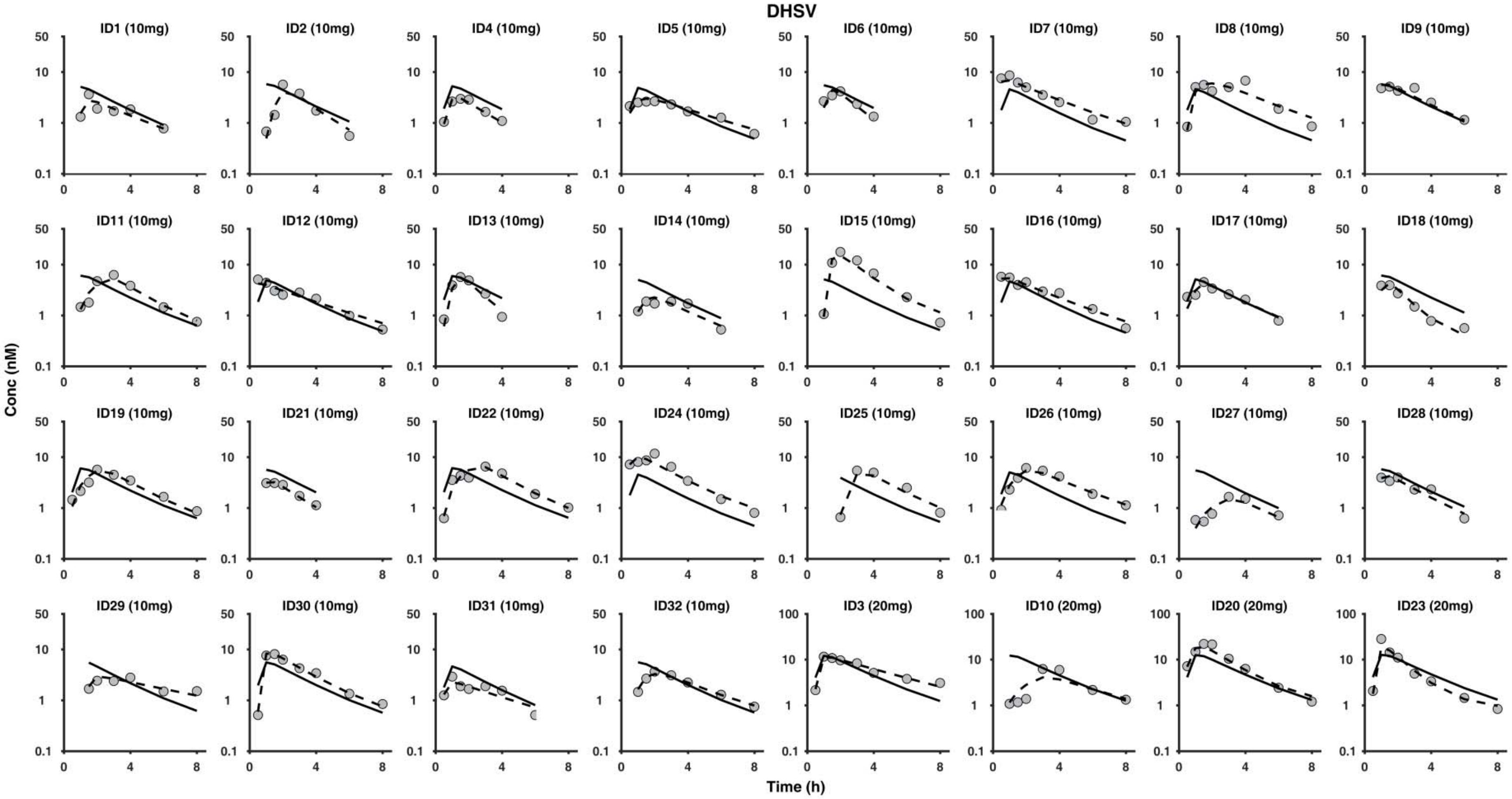

References

1 Beal SL (2001) Ways to fit a PK model with some data below the quantification limit. J Pharmacokinet Pharmacodyn 28 (5): 481-504

2 Karlsson MO, Beal SL, Sheiner LB (1995) Three new residual error models for population PK/PD analyses. J Pharmacokinet Biopharm 23 (6): 651-672

3 Tsamandouras N, Dickinson G, Guo Y, Hall S, Rostami-Hodjegan A, Galetin A, Aarons L (2014) Identification of the Effect of Multiple Polymorphisms on the Pharmacokinetics of Simvastatin and Simvastatin Acid Using a Population-Modeling Approach. Clin Pharmacol Ther 96 (1): 90-100
